# Source, Distribution and Potential Risk of Antimony in Water and Sediments of Danjiangkou Reservoir: Impact from Dam

**DOI:** 10.3390/ijerph191912367

**Published:** 2022-09-28

**Authors:** Haihua Zhuo, Yunli Wu, Yunbing Liu, Jie Xu, Xueqin Guo, Jie Chen, Xuejiao Ouyang

**Affiliations:** Yangtze River Basin Ecological Environment Monitoring and Scientific Research Center, Yangtze River Basin Ecological Environment Supervision and Administration Bureau, Ministry of Ecology and Environment, Wuhan 430010, China

**Keywords:** Danjiangkou Reservoir, antimony, source, distribution, risk

## Abstract

Danjiangkou Reservoir is the water source of the mid-route of the South-to-North Water Transfer Project. The source, distribution and potential risk of antimony in its water and sediments are rarely reported. In this study, symmetrical investigation results demonstrated that the concentration of antimony in the Han sub-reservoir and water in front of the dam fluctuated at about 0.9 mg L^−1^, while it was relatively higher and increased with the distance from the dam in Dan sub-reservoir water, with an annual average of 0.93~3.15 mg L^−1^. In recent years, the concentration of antimony in the Danjiangkou Reservoir showed a downward trend, and the difference between the Han and Dan sub-reservoirs decreased significantly. The antimony in the sediments in the reservoir was primarily derived from the inflowing rivers, and it was higher in the Dan sub-reservoir than in the Han sub-reservoir. The concentration of antimony in the water in the reservoir was considerably higher than the background value in the watersheds, indicating that there is an external input with decreasing input intensity. The content of antimony in the sediments in the reservoir and its inflow rivers was substantially higher than the background value of watersheds, indicating that there is a certain degree of enrichment. In addition, the antimony mining industry in the water source area poses a risk to the water safety of the reservoir. Antimony is not a conventional pollutant. Consequently, the collection of antimony monitoring results is a challenging task. Additionally, this study fills the gap in regional antimony research. Furthermore, the ecological risk assessment of antimony in China is still in its infancy. Unquestionably, the study of the temporal and spatial distribution of antimony concentration will be beneficial for the protection of water sources in relevant regions.

## 1. Introduction

Antimony is frequently utilized in flame retardants, battery alloys, plain bearings and solders, despite its potential toxicity and carcinogenicity [1,2]. The United States Environmental Protection Agency (USEPA) and the European Union Basel Convention list antimony as a priority control pollutant and hazardous waste, respectively [3,4]. Compared with conventional pollutants such as lead, mercury and cadmium, antimony has rarely attracted public attention, but this does not affect the strong toxicity of excessive antimony. China is a large country characterized by antimony resources and production, with reserves and production ranking first in the world [5]. Antimony deposits have a tendency of regional concentration in spatial distribution, and the deposits are principally concentrated in Hunan, Guangxi, Tibet, Guizhou, Yunnan, Gansu and other 19 provinces [6,7]. China has been far ahead of other major antimony resource countries in terms of antimony production, with an average annual output of approximately 100,000 tons in 2017, accounting for 73% of the world’s total output [8,9]. Nevertheless, China has contributed to some regional water and soil pollution in recent years with its extensive industrial utilization of antimony and large-scale antimony mining [7,10]. Worse still, there have even been some significant pollution incidents, such as the Gansu Longxing antimony industry limited liability company tailings leakage incident at the end of 2015 [11].

As the water source of the South-to-North Water Diversion Project, the Danjiangkou Reservoir plays a critical strategic role in safeguarding the economic and social sustainability of China [12]. The reservoir collects surface water from more than 40 counties and cities in Hubei, Henan and Shaanxi provinces, and its water quality is closely related to the safety of drinking water for a large number of people [13]. Part of the Danjiangkou water source area is in Kuncang-Qinling antimony metallogenic belt [14,15]. This metallogenic belt includes the Xunyang Mansion deposit in Shaanxi province, the Danfeng Caiao deposit and Lu Dahe Gully–Qingjia Gully deposit in Henan Province and the Yunxi Gaoqiaopo deposit in Hubei Province [16,17]. Most of these antimony ores have been mined to some extent [18,19]. Due to the mining and beneficiation activities of antimony ore in recent years, the water quality of Danjiangkou water source areas has posed a certain risk [16,20]. Notably, in November 2021, a mine leakage accident occurred because of the abandoned antimony mine in the Wulichuanhe River Basin, a tributary of the upper reaches of the Laoguanhe River. At present, the public is primarily concerned with the water quality and water ecology of Danjiangkou Reservoir, and studies on heavy metals are relatively scarce [21]. Intense research concentrates mainly on copper, lead, zinc, cadmium, chromium and other elements, whereas there are scant reports on the antimony content distribution in water and sediments.

In this study, some typical areas were selected in Danjiangkou Reservoir, and the antimony contents in water and sediments were investigated. The influence of the dam on the source and distribution of antimony concentrations was highlighted. Furthermore, the potential risk was assessed via a long time-scale monitoring and statistics, and then the change tendency in the future was analyzed in order to provide the basic information for the supervision of antimony pollution in Danjiangkou Reservoir.

## 2. Materials and Methods

The Danjiangkou Water Control Project is located in Danjiangkou City, Hubei Province, China, 800 m below the confluence of the Han River and the Danjiang River. It has extensive benefits, including flood control, power generation, water diversion, irrigation, shipping and aquaculture.

**Figure 1:**
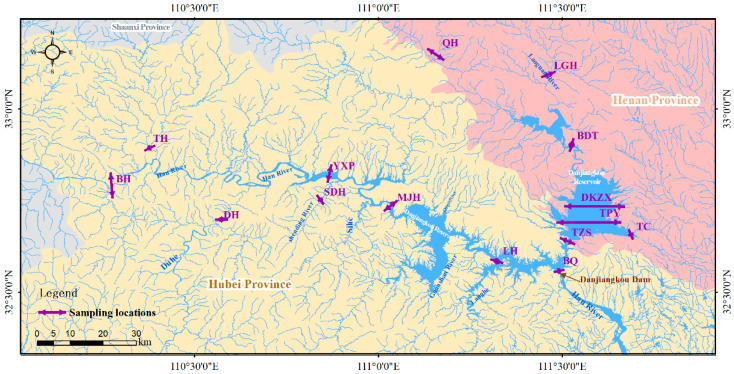
Sampling locations for the antimony concentrations in the water and sediments in Danjiangkou Reservoir. Reservoirs located in Henan Province are called the Dan sub-reservoir, and those located in Hubei Province are called the Han sub-reservoir.

The Danjiangkou water source area involves a large area. In this study, the sections of Taocha (TC), Dan sub-reservoir center (DKZX), Taizishan (TZS), above the dam (BQ), Baidutan (BDT), Mojiahe River (MJH), Yangxipu (YXP), Du River (DH), Laoguan River (LGH), Tianhe (TH), Qihe (QH), Taipingyang (TPY), Langhe (LH), Baihe (BH) and Shending River (SDH) were selected. All sampling points are located in the control section set by the state. When setting the control section, it is required to obtain the most representative samples with fewer monitoring points, so as to comprehensively, truly and objectively reflect the water environment quality and the temporal and spatial distribution and characteristics of pollutants in the region. When large tributaries converge in a river reach, sampling should be undertaken upstream of the tributaries at the confluence point, and additional sampling points should be added in waters with a sensitive water environment. Figure 1 portrays the sample collection section.

The antimony concentration was determined by the water conservancy industry standard of the People’s Republic of China (SL 394.1-2007), which is an authoritative test method recognized in China, whose test outcomes were consistent with the cerium sulfate titration method. Each liter of collected water sample was treated with 10 mL of concentrated HNO_3_ and stored in cold storage, and the test was completed within 30 days. After removing the pebbles and animal and plant residues in the collected sediment samples, the sediment samples were well mixed and divided into four fractions for air drying; then they were screened and digested for detection.

After confirming and removing outliers from the data, Microsoft Office Excel 2010 was utilized for processing, statistics, analysis and drawing.

## 3. Results and Discussion

**Figure 2:**
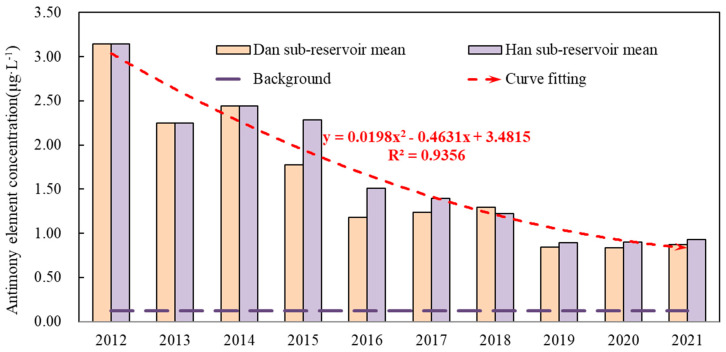
Trend of antimony concentrations in Danjiangkou Reservoir water. The red dotted line in the figure is the fitting curve of the mean antimony concentration in the Han sub-reservoir and Dan sub-reservoir as a function of time.

The concentration statistics of antimony elements in various water bodies of Danjiangkou Reservoir are presented in Table 1 and Figure 2. The antimony concentration in Han sub-reservoir (YXP and MJH) water was relatively low, fluctuating at about 0.9 μg L^−^^1^. The antimony concentration at BQ was basically the same as that in Han sub-reservoir water, with an average of 0.92 μg L^−1^ in each year. The mean concentration of antimony in the TZS of the Dan sub-reservoir (the boundary section between Hubei and Henan Province) was 1.37 μg·L^−1^. The mean of the TC water in the main channel of the mid-route of the South-to-North Water Transfer Project was 1.30 μg L^−1^. The mean of DKZX water was 1.53 μg L^−1^, while it was 3.91 μg·L^−1^ in BDT water. It was discovered that the amount of antimony in Han sub-reservoir water was comparatively lower, whereas the concentration of antimony in Dan sub-reservoir water generally increased from proximity to distance from Danjiangkou dam. The antimony concentrations of the seven monitoring sites shown in Table 1 all illustrated a decreasing trend. The results of univariate linear fitting depicted that TC and DKZX portrayed a significant decrease (*p* < 0.05), the remaining five locations all exhibited fluctuating declines (*p* > 0.05), and the annual decrease rate of 7 points was 10–50%. The antimony concentration in TZS, TC and DKZX water revealed a declining trend from 2012 to 2019, after which it remained relatively stable. Despite the fact that the antimony concentration in Han sub-reservoir (YXP) water was still lower than that in Dan sub-reservoir, the difference had diminished dramatically, and there was little difference between the two reservoirs from 2020 to 2021.

The background values of elements are extremely important for examining the geochemical behavior and properties of elements [22,23,24,25]. The background values of antimony in the Yangtze River system and Han River system were 0.13 μg L^−1^ and 0.12 μg L^−1^, and the ranges were 0.04 to 0.41 μg L^−1^ and 0.04 to 0.81 μg L^−1^, respectively [26,27]. The antimony concentrations in all sites of Danjiangkou Reservoir were significantly higher than the background values in the Yangtze River system and the Han River system, and only the measured values in some years and sites were slightly lower than the upper limit of background values. Some researchers have demonstrated that the water body of Danjiangkou Reservoir received external pollution input [28]. Notwithstanding, this study indicated that the intensity of antimony input is decreasing. A portion of the Danjiangkou water source region was located within the Kuncang-Qinling antimony metallogenic belt, and there were numerous antimony mining operations in the vicinity of the Danjiangkou Reservoir and along the major inlet rivers. Since the start of the South-to-North Water Transfer Project, the relevant mining enterprises in the Danjiangkou water source area had been strictly purged. Then, no new mining activities were added, and ore washing and beneficial activities were also suspended. In addition, no new pollution sources were added. This is likely the predominant reason for the decline in antimony concentration in Danjiangkou Reservoir water over the past few years. Although these mining enterprises had been rectified, more than 300 tailing ponds still existed in the reservoir area, and some abandoned antimony mines left mine slag that still discharged high concentrations of antimony-containing wastewater under certain external forces (e.g., precipitation); for example, in November 2021, the antimony pollution event in the Wulichuan River, a secondary tributary of the Danjiang River, posed a serious threat to reservoir water quality. Due to the dam being raised as part of the project, the reservoir’s storage will increase 157 m to 170 m, adding 305 km^2^ of water storage area [29]. A huge proportion of antimony from the soil is transferred to the reservoir, raising the likelihood of contamination.

From the perspective of protecting human health, the limit value of total antimony is 5 μg·L^−1^ in the current Surface Water Environmental Quality Standard (GB 3838-2002) and Sanitary Standard for Drinking Water (GB 5749) [30]. Monitoring results based on this criterion confirmed that in 2012 there was an exceedance of the standard allowable limit at the BDT site, while no exceedances were documented at other times and locations. Currently, there is a lack of antimony water quality benchmarks for the protection of freshwater aquatic organisms in China. Despite the fact that relevant scholars have conducted relevant research, they genuinely think that if only for the purpose of protecting aquatic organisms, the current standards suffer from “overprotection” [31]. Antimony can exist in nature in a variety of oxidized forms (−3, 0, +3, +5). Inorganic antimony is primarily found in trivalent and pentavalent forms in organisms and environments, and organic antimony compounds are chiefly found in the form of mono- to trimethyl antimony; the toxicity of different forms of antimony varies significantly [32]. The Danjiangkou Reservoir is the water source of the mid-route of the South-to-North Water Transfer Project. In view of its significant strategic position, it is suggested that the relevant departments should organize a traceability survey of antimony in the water source area as soon as possible and determine the amount and form of antimony element in the water and organisms. On this basis, the aquatic biological water quality benchmark and antimony exposure risk assessment suitable for antimony in Danjiangkou water source area should be established to provide a scientific basis and reference for risk management of antimony pollution in the regional water environment.

**Figure 3:**
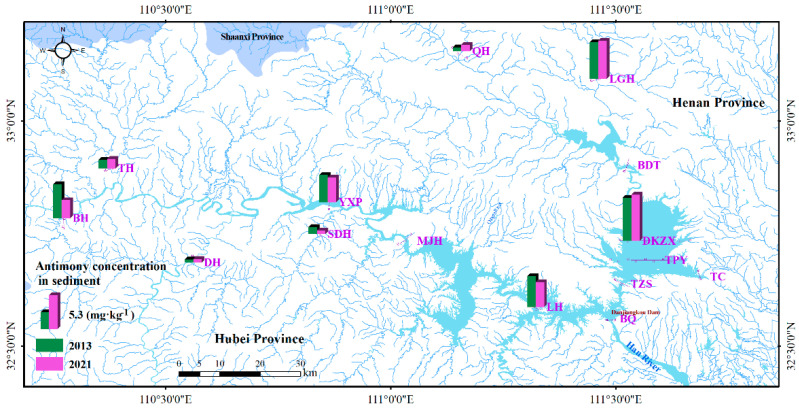
Distribution of antimony contents in Danjiangkou Reservoir sediments.

Monitoring of sediments was performed in the estuary of a number of major inflow rivers and main sites in Danjiangkou Reservoir. To simplify the analysis, 2013 and 2021 were employed as representative years, and the monitoring results are shown in Figure 3. The South-to-North Water Diversion Project officially started water delivery in 2014, so 2013 is a representative year before water transfer. In 2019, the South-to-North Water Diversion Project began to transfer water at full capacity and stability, so 2020 and 2021 are representative years after full-load water transfer. Among the rivers flowing into the Han sub-reservoir, the content of antimony in sediments of BH in the Han River was 5.36 mg·kg^−1^ and 2.84 mg·kg^−1^ in 2013 and 2021, respectively. The contents of antimony in the river estuaries of TH, DH and SDH were relatively lower. Among the rivers flowing into the Dan sub-reservoir, the contents of antimony in QH estuaries were lower, while the content of antimony in sediments was higher in LGH estuaries, with values of 5.75 mg·kg^−1^ and 6.03 mg·kg^−1^, respectively. In 2013 and 2021, the sediment content of antimony in the sites such as YXP in Han sub-reservoir and below the LGH estuary was around 4.0 mg·kg^−1^, while the sediment concentration of sites such as TZS and Pacific in Dan sub-reservoir was obviously higher than that of the Han sub-reservoir, with the measured value above 6.78 mg·kg^−1^. These results indicated that the antimony in Danjiangkou Reservoir sediments originated chiefly from some of the confluent rivers and that the antimony content in Dan sub-reservoir sediments may be higher than that in Han sub-reservoir sediments. Since the study area is positioned in the Kuncang-Qinling antimony metallogenic belt and there were antimony ore mining activities such as washing and beneficiation in the early stages of the area’s development, antimony may have entered the sediments carried by rivers.

Taking the sediment background of the Han River (2.0 mg·kg^−1^) as a reference, the sediments in the Han River, LGH and reservoir were obviously 2 to 3 times higher, while they were more significantly higher taking the Yangtze River system (1.1 mg·kg^−1^) or the national water system background value (0.7 mg·kg^−1^) as a reference. Sediment is a meaningful location and the fundamental storage site for heavy metals in water bodies, as well as a potential receptor and source of pollution [33]. Under certain conditions, heavy metals in sediments and water bodies undergo migration and transformation, and heavy metal pollutants in water bodies settle into sediment with suspended sediment, while sediment releases heavy metals into water under conditions such as water body disturbance and changes in redox conditions [34,35]. The concentration of antimony in different waters of Danjiangkou Reservoir has decreased significantly in recent years (or is generally at a low level), whereas its concentration in sediments has not changed (although the evaluation of such spatial and temporal trends may not be very accurate due to the limitations of measurement points and the number of measurements). Further investigation is required to comprehend the sources of sediment in Danjiangkou Reservoir, the processes by which antimony elements move through water and transform into other elements, the aquatic biological water quality, and the sediment benchmark studies that are appropriate for antimony in the Danjiangkou water source area. The Danjiangkou Reservoir serves as a source of water for the entire country, and this migration and transformation could endanger aquatic life and potentially harm the water quality and security of water transfer.

## 4. Conclusions and Recommendations

(1) The concentration of antimony in the water of the Han sub-reservoir and in front of the dam in Danjiangkou Reservoir was relatively low, fluctuating around 0.9 mg·kg^−1^. The concentration of antimony in the water of the Dan sub-reservoir was relatively high, and its concentration generally increased with the distance from Danjiangkou dam. In recent years, the reservoir’s antimony concentration has declined significantly, and the gap between the concentrations in the Dan and Han sub-reservoirs has remarkably narrowed. The content of antimony in Danjiangkou Reservoir sediments mainly originated from some of the confluent rivers. The distribution of antimony in water bodies was similar, with sediments in the Dan sub-reservoir containing a greater antimony content than those in the Han sub-reservoir.
(2) The overall antimony element concentration in Danjiangkou Reservoir water was significantly higher than the background values in the Yangtze River system and Han River system, which indicated the existence of exogenous input. Nonetheless, the intensity of input has decreased in recent years. The contents of antimony in the sediments of Danjiangkou Reservoir and the rivers that feed it, including the Han River and the Laoguan River, were significantly higher than background levels found in sediments from the national and Yangtze River systems, implying some degree of enrichment.
(3) Numerous inferences in this study must be further confirmed in the absence of auxiliary survey data, and we draw attention to antimony contamination. Given that antimony is toxic and carcinogenic, this study served as the foundation for appraising the risk of the antimony mining industry of a certain scale existing in some areas of the Danjiangkou water source area, and it showed that antimony mining and tailing ponds posed certain risks to the water safety of the Danjiangkou Reservoir.

## Figures and Tables

**Table 1:** Statistics of antimony monitoring results in Danjiangkou Reservoir water bodies (μg L^−1^).

Projects	Han Sub-Reservoir		BQ	Dan Sub-Reservoir				Mean of Dan Sub-Reservoir	Mean of All Reservoirs
	YXB	MJH		**TZS**	**TC**	**TPY**	**BDT**		
2012	-	-	-	1.90	2.14	-	5.40	3.15	3.15
2013	-	-	-	2.70	0.40	2.90	3.00	2.25	2.25
2014	-	1.19	1.52	1.58	2.32	2.26	5.76	2.44	2.44
2015	-	0.88	0.64	0.82	1.52	2.00	4.79	2.28	1.78
2016	-	0.45	0.57	0.71	1.38	1.46	2.50	1.51	1.18
2017	-	0.90	0.95	1.09	1.21	1.31	1.99	1.40	1.24
2018	1.50	-	-	1.22	1.44	1.02	-	1.23	1.30
2019	0.75	-	-	-	0.70	1.09	-	0.90	0.85
2020	0.70	-	-	-	0.90	0.90	-	0.90	0.83
2021	0.70	-	-	0.91	1.01	0.86	-	0.93	0.87
AVG	0.91	0.86	0.92	1.37	1.30	1.53	3.91	1.70	1.59
S.D.	0.39	0.30	0.43	0.67	0.60	0.71	1.61	0.78	0.79
Background values	Hanjiang River 0.12 (range 0.04 to 0.41)								
	Yangtze River 0.13 (range 0.04 to 0.81)								

## Data Availability

Not applicable.
